# P-991. Rapid Molecular Diagnostics in Hospital-Acquired and Ventilator-Associated Pneumonia : Awareness and Practices Among Infectious Diseases Trainees

**DOI:** 10.1093/ofid/ofaf695.1190

**Published:** 2026-01-11

**Authors:** Sandeep Rao Kordcal, Md Tariq Maula, Indrashekhar Prasad, Tanu Sagar

**Affiliations:** All India Institute of Medical Sciences, New Delhi, Delhi, India; All India Institute of Medical Sciences, New Delhi, Delhi, India; All India Institute of Medical Sciences, New Delhi, Delhi, Delhi, India; All India Institute of Medical Sciences, New Delhi, Delhi, Delhi, India

## Abstract

**Background:**

Multiplex PCR panels for respiratory specimens can lead to rapid detection of bacterial pathogens and clinically significant antimicrobial resistance (AMR) genes in patients with suspected hospital-acquired pneumonia(HAP)/ventilator-acquired pneumonia (VAP). This promises to improve diagnostic stewardship and ultimately impact patient outcomes.

**Methods:**

We conducted an anonymized, structured survey among 24 infectious diseases resident doctors (trainees) at a tertiary care academic center. The questionnaire assessed knowledge of a multiplex PCR panel, BioFire FilmArray Pneumonia Plus Panel , its clinical utility, and perceived barriers to optimal use.

**Results:**

A majority (96%) of respondents recognized the rapid turnaround time of multiplex PCR (< 2 hours). However, only 42% correctly identified that these panels detect bacterial but not fungal pathogens. Although 92% agreed that culture remains essential even after multiplex PCR testing, only 62% expressed confidence in modifying empirical therapy based solely on multiplex PCR results. Despite negative multiplex PCR results, 58% preferred continuing empirical antibiotics in critically ill patients, citing clinical judgment and possibility of missing infections. Anxiety about missed infections despite negative multiplex PCR results was reported by 62% of trainees, highlighting a significant emotional barrier to antibiotic de-escalation. While 71% agreed that multiplex PCR enhanced their confidence in antibiotic de-escalation, 88% reported occasionally overriding multiplex PCR findings based on clinical suspicion. Consultation with microbiologists during interpretation remained infrequent.

Barriers to optimal use included institutional culture (12%) and limited confidence in test results (8%).

**Conclusion:**

Among ID trainees, awareness of multiplex PCR panel utility is high, but certain knowledge gaps persist. Clinical hesitation and inertia needs to be addressed as new diagnostic modalities roll out. While the optimal approach to antibiotic escalation and de-escalation continues to evolve, structured educational interventions and evidence-based reforms in prevailing practices can help optimize the use of molecular diagnostics in HAP/VAP management.

**Disclosures:**

All Authors: No reported disclosures

